# Digital Transformation of Public Health for Noncommunicable Diseases: Narrative Viewpoint of Challenges and Opportunities

**DOI:** 10.2196/49575

**Published:** 2024-01-25

**Authors:** Onicio Leal Neto, Viktor Von Wyl

**Affiliations:** 1 Department of Computer Science ETH Zurich Zurich Switzerland; 2 Global Health Institute Mel & Enid Zuckerman College of Public Health University of Arizona Tucson, AZ United States; 3 Department of Epidemiology and Biostatistics Mel & Enid Zuckerman College of Public Health University of Arizona Tucson, AZ United States; 4 Institute for Implementation Science in Health Care University of Zurich Zurich Switzerland; 5 Epidemiology, Biostatistics & Prevention Institute University of Zurich Zurich Switzerland

**Keywords:** digital public health, artificial intelligence, non-communicable diseases, digital health, surveillance, well being, technological advancement, public health efficiency, digital innovation

## Abstract

The recent SARS-CoV-2 pandemic underscored the effectiveness and rapid deployment of digital public health interventions, notably the digital proximity tracing apps, leveraging Bluetooth capabilities to trace and notify users about potential infection exposures. Backed by renowned organizations such as the World Health Organization and the European Union, digital proximity tracings showcased the promise of digital public health. As the world pivots from pandemic responses, it becomes imperative to address noncommunicable diseases (NCDs) that account for a vast majority of health care expenses and premature disability-adjusted life years lost. The narrative of digital transformation in the realm of NCD public health is distinct from infectious diseases. Public health, with its multifaceted approach from disciplines such as medicine, epidemiology, and psychology, focuses on promoting healthy living and choices through functions categorized as “Assessment,” “Policy Development,” “Resource Allocation,” “Assurance,” and “Access.” The power of artificial intelligence (AI) in this digital transformation is noteworthy. AI can automate repetitive tasks, facilitating health care providers to prioritize personal interactions, especially those that cannot be digitalized like emotional support. Moreover, AI presents tools for individuals to be proactive in their health management. However, the human touch remains irreplaceable; AI serves as a companion guiding through the health care landscape. Digital evolution, while revolutionary, poses its own set of challenges. Issues of equity and access are at the forefront. Vulnerable populations, whether due to economic constraints, geographical barriers, or digital illiteracy, face the threat of being marginalized further. This transformation mandates an inclusive strategy, focusing on not amplifying existing health disparities but eliminating them. Population-level digital interventions in NCD prevention demand societal agreement. Policies, like smoking bans or sugar taxes, though effective, might affect those not directly benefiting. Hence, all involved parties, from policy makers to the public, should have a balanced perspective on the advantages, risks, and expenses of these digital shifts. For a successful digital shift in public health, especially concerning NCDs, AI’s potential to enhance efficiency, effectiveness, user experience, and equity—the “quadruple aim”—is undeniable. However, it is vital that AI-driven initiatives in public health domains remain purposeful, offering improvements without compromising other objectives. The broader success of digital public health hinges on transparent benchmarks and criteria, ensuring maximum benefits without sidelining minorities or vulnerable groups. Especially in population-centric decisions, like resource allocation, AI’s ability to avoid bias is paramount. Therefore, the continuous involvement of stakeholders, including patients and minority groups, remains pivotal in the progression of AI-integrated digital public health.

## Introduction

The SARS-CoV-2 pandemic saw the broad roll out and application of the first digital public health intervention at scale: digital proximity tracing (DPT) [1]. The general idea of DPT smartphone apps consists of leveraging the capabilities of standard Bluetooth sensors to trace proximity contacts and to notify the exposed individuals of past proximity encounters in case of a confirmed SARS-CoV-2 infection. The development and wide-ranging use of DPT apps have been a success story on several different levels. Their speedy development and release early in the pandemic reflects the prosperous international collaboration and backing by many relevant organizations such as the World Health Organization or the European Union.

To maintain the momentum for digital public health, it will be important to extend the digital transformation of public health in the domain of noncommunicable diseases (NCD). Prior to the pandemic, approximately 80% of all health care expenditures and 63.8% of the premature loss of disability-adjusted life years were associated with NCDs [2]. The challenges, but also opportunities, of NCD digital public health are quite different from the public health response to mitigate infectious diseases such as SARS-CoV-2. This perspective paper will start with an overview of current definitions of digital public health. Furthermore, in addition to requirements for digital transformation, we will explore different aspects that can guide the identification of potential use cases for digital public health applications in NCDs. The paper will close with an outlook on challenges and conditions to make further inroads on digitally transforming NCD public health.

## Key Functions of Public Health

Public health is defined as the science and practice to establish conditions so that people can live healthy lives and make healthy choices. It draws on methods and knowledge of multiple disciplines such as epidemiology, medicine, psychology, law, social sciences, and many others [3]. Textbox 1 illustrates the essential public health functions that should be implemented by jurisdictions to achieve equitable health and health system access. These functions can broadly be grouped into “Assessment,” “Policy Development,” “Resource Allocation,” “Assurance,” and “Access.”

The framework emphasizes that public health involves more than just securing access to or ensuring high-quality medical care for individuals and an adequate health care workforce. Generally, public health also has a strong focus on communities and touches on the foundations for healthy living (eg, living conditions, education, or economic aspects), as well as population-based preventive measures (eg, childhood vaccinations).

**Institutional framework of essential public health functions.**

**Assessment**

- Assess and monitor population health status and well-being, factors that influence health and its social determinants, community needs and assets, and health system performance and impact.
- Investigate, diagnose, and address health problems and hazards affecting the population, including public health surveillance and control and management of health risks and emergencies.
- Promotion and management of health research and knowledge.

**Policy development**

- Communicate effectively to inform and educate people about health, factors that influence it, and how to improve it.
- Strengthen, support, and mobilize communities and partnerships to improve health.
- Create, champion, and implement policies, plans, and laws that impact health.
- Use legal and regulatory actions designed to improve and protect the public’s health.

**Assurance**

- Assure an effective system that enables equitable access to the individual services and care needed to be healthy.
- Build and support a diverse and skilled public health workforce.
- Improve and innovate public health functions through ongoing evaluation, research, and continuous quality improvement.
- Build and maintain a strong organizational infrastructure for public health.
- Development and implementation of health policies and promotion of legislation that protects the health of the population.
- Social participation and social mobilization, inclusion of strategic actors, and transparency.

**Resource allocation**

- Development of human resources for health.
- Ensuring access to and rational use of quality, safe, and effective essential medicines and other health technologies.
- Efficient and equitable health financing.
- Equitable access to comprehensive, quality health services.

**Access**

- Equitable access to interventions that seek to promote health, reduce risk factors, and promote healthy behaviors.
- Management and promotion of interventions on the social determinants of health.

Note: The framework is based on The Public Health National Center for Innovation [4] and Pan-American Health Organization [5].

## Levels of Digital Changes

### Overview

In health care and public health the levels to which specific processes and workflows, can be turned “digital” varies drastically. These levels are commonly described as digitization (moving from analogic to digital data collection), digitalization (integration of digital tools into established processes and workflows), and digital transformation (redesigning processes and workflows around digital tools). For the remainder, we will use the more neutral term “digital change,” which is intended to encompass all 3 levels.

Digital change attempts to achieve an impact on public health and health care by enabling better, more efficient, and more cost-effective public health interventions [6]. Thus it mirrors the triple aim of health care: improving patient outcomes, enhancing patient experiences, and reducing health care costs [7]. As such, digital change may also help to steer public health’s directions toward the goal of the 4Ps: preventive, predictive, personalized, and participatory [8]. By harnessing the -omics revolution, but also digital technologies such as wearables, mobile apps, and artificial intelligence (AI), health care providers can deliver preventive care by identifying potential health issues before they become severe. Predictive analytics can help identify patients at risk of developing certain conditions, allowing for earlier intervention and treatment. Personalized medicine can also be delivered through digital tools that analyze patient data to create tailored treatment plans. Finally, participatory health care allows for greater collaboration among communities, providers, and individuals, enabling these groups to take a more active role in managing their health. Consumers and patients are increasingly empowered to collect their own data and access medical information, thus gaining confidence, and claiming a role in health decision-making.

### Conditions for Successful Digital Change in Health Care and Public Health

On the technical side, system readiness, interoperability, technical application programming interfaces, and nontechnical interfaces are crucial for implementing digital public health. System readiness ensures that digital technologies are compatible with existing health care systems and infrastructure [9,10]. Interoperability is essential to enable different systems to work together seamlessly, enabling for exchange of information and data between different platforms [11,12]. Interoperability also fosters the ability to collect and analyze vast amounts of health data in real time, thus allowing public health practitioners, policy makers, and citizens to gain valuable insights into health outcomes, treatment effectiveness, and disease trends [13]. As such, AI-driven analytics may also foster participation and personalization, as postulated by P4 [8]. However, ensuring that data analysis is unbiased and AI fairness is maintained is essential to avoid automating inequalities driven by AI modeling [14,15].

A further critical element in digital change routes is the scalability of technical systems and processes as health care systems look to scale their operations, widen care to more patients, and enhance the democratization of health care and public health [16]. Cloud-based platform solutions can help those public systems achieve scalability while reducing costs and improving efficiency as well as reducing disparities in public health [17-19]. Scalability also pertains to practices that involve humans-in-the-loop. Therefore, preparing the health care and public health workforce for digital changes is an important avenue to fill the gaps in skills and digital literacy in the next generation of public health practitioners, hence ensuring that health care systems can fully leverage the benefits of digital technologies [20-23]. Training programs and courses should focus on various disciplines and domains, including the Internet of Things, data analysis, data stewardship, and AI. Other essential domains for training programs and courses include cybersecurity, data privacy, and regulatory compliance where health care operators must be trained to ensure that they can protect patient data and comply with relevant regulations and legislation. Training programs should also cover soft skills such as communication, teamwork, and problem-solving, ensuring that health care providers can work effectively in interdisciplinary teams and collaborate with other stakeholders.

Creating a favorable legal and ethical context is critical for successful digital changes in public health. Legal frameworks must provide clear guidance on data privacy, security, and ownership issues [24,25]. In addition to legal and ethical considerations, financial factors also play a significant role in digital transformation in public health. Advantageous financial incentives can encourage health care providers to invest in digital technologies, enabling them to deliver better, more efficient, and more cost-effective care. Reimbursement and remuneration systems must also be adapted to account for incentivizing the development and use of digital technologies, ensuring that technology and service providers are fairly compensated for these technologies [26]. These models must also consider the value of these technologies, such as improved health outcomes, reduced health care costs, and increased user (ie, citizens or patients) satisfaction [27,28].

Yet, it will also be essential to plan for unexpected events or circumstances that could derail digital transformation efforts. By establishing a robust risk management plan and building flexibility into digital transformation initiatives, health care providers can minimize the impact of unforeseen events and ensure that their digital transformation efforts continue to deliver value and benefits to patients and health care providers alike [29-33].

### Digital Change + Public Health = Digital Public Health?

The explicit connection between “digital” and “public health” is quite recent and well summarized by Iyamu et al [34], who trace “digital public health” back to a report by Public Health England from 2017 [35]. This report essentially calls for a reimagination of public health using digital tools, thus calling for a digital transformation of public health, a view that has recently been shared by representatives of the European Public Health Association [34]. As part of the transformation process, some authors suggest not only focusing on the technical possibilities but also looking for inspiration from the start-up culture of the tech industry, with a stronger emphasis on experimenting and adapting (pivoting) rather than waiting until scientific evidence has solidified. Other definitions describe digital public health as an “asset” toward achieving traditional public health goals, for instance by leveraging new data, methods, and work processes that have been given rise by digitization [36]. Therefore, these existing definitions appear to differ mainly in the meaning and role of “digital” on the digitalization-digital transformation spectrum, as already pointed out by Iyamu. But all authors agree that a consequential digital public health goes way beyond the digitization of existing services and interventions—they require rethinking and redesigning full health care delivery and public health services pathways.

**Figure 1:**
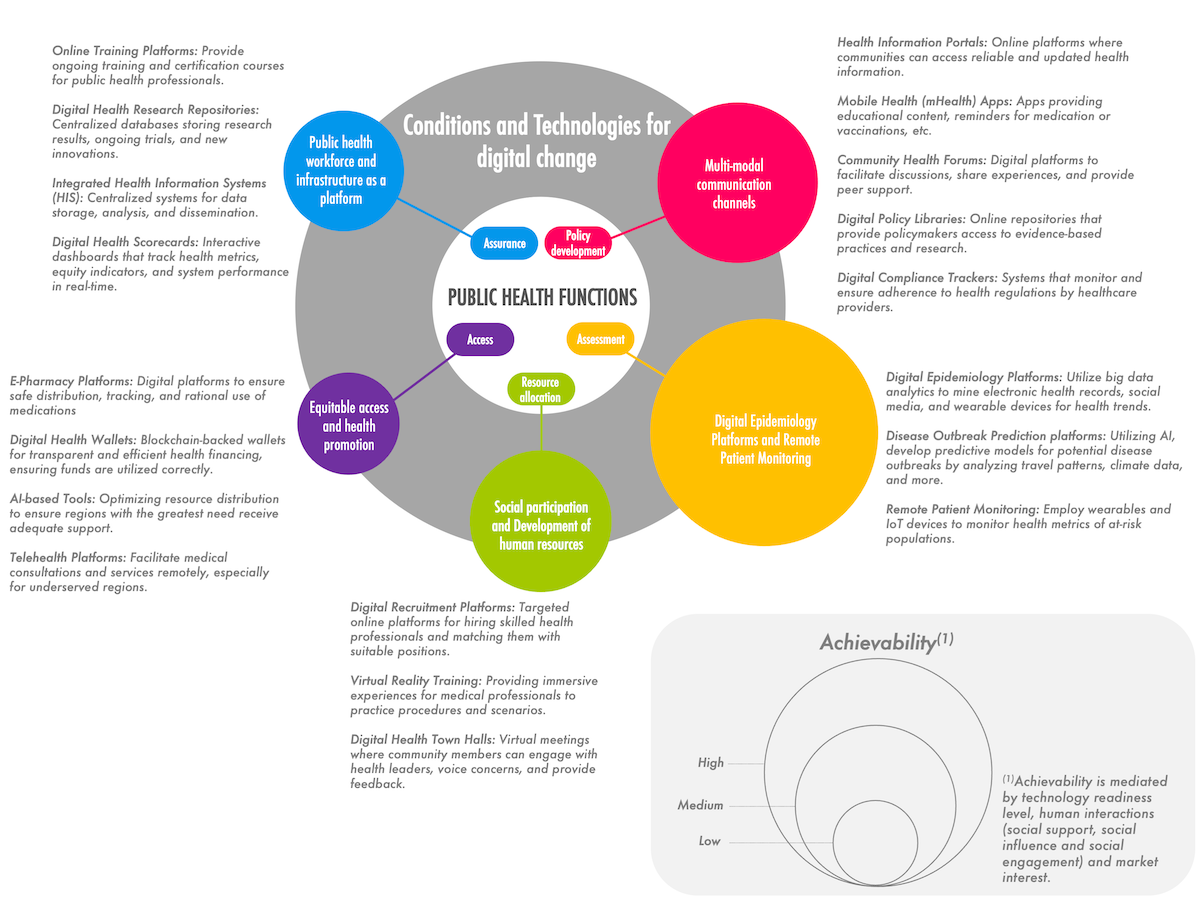
Conditions and technologies for digital change and concrete examples based on the public health functions. AI: artificial intelligence.

Considering the framework of essential public health functions (Textbox 1), there are likely some domains that lend themselves more easily to a digital change, because digital data acquisition and analysis are at their core. Matching public health functions with suitable goals and purposes of digital change processes is critical for reaping the benefits of digital technologies. One of the key areas where digital transformation can be leveraged is in complex data analysis [37,38]. By contrast, the “digitalization or digital transformation question” is less easily resolved for functions such as building a sufficient health care workforce or equitable health care access. This is visualized in Figure 1 as conditions and technologies for digital change. In the figure, the “Public Health Functions” (Textbox 1) are depicted in the inner circle, and possible examples of digitally changed essential public health function processes are shown in the outer circle. The size of the circles in the outer circle illustrates our broad assessment of the achievability of digital change for specific public health functions, by taking the bespoke conditions for successful change into account, namely technical readiness, reliance on human interactions, or market interest in existing public health function processes. In general, processes relying on data collection and analysis or digitally facilitated mass communication lend themselves more easily to digital changes, while the creation and maintenance of an adequate workforce for health care and public health will likely continue to depend on human factors in the foreseeable future.

### Goal and Purpose of Digital Change

To make further inroads toward digital change in public health functions and NCD public health in particular, it seems sensible to distinguish between the goal and purpose of making these functions and processes more digital. By goal, we mean the high-level public health goals of broad health care quality, access, efficiency, and equity of health promotion and health care provision, which can be fostered by digital changes. For instance, in light of the shortage of mental health care providers in many settings, apps for mental health assessments may ease access to at least some basic level of mental health care. Indeed, 24 of the 54 reimbursable digital health apps [39] (as of August 30, 2023) in Germany address mental health challenges including stress, burnout, or depression.

By contrast, we consider the purpose of digital change as improvements in the public health or health care service delivery process by digital change. These process attributes can, for example, be defined in analogy to the “triple aim” of health care innovation whether digital change renders the process of providing and delivering existing or novel public health measures and health care in a more effective, efficient, or user-friendly manner.

An alternative view on process attributes that are influenced by digital changes is offered by Peter Diamandis’ 6Ds of exponential technologies [16,40]. This concept identifies 6 categories of process attributes—digitization, deception, disruption, demonetization, dematerialization, and democratization—which also outlines the desired trajectory of a digital change process. Initiating digital changes in the health care sector may initially elicit resistance in some actors, who may initially respond with incredulity about the potential benefits of these changes in the short term. As the proposed innovation begins to ramp up, it reduces inefficiencies and optimizes current systems, disrupting the same old processes. Demonetization occurs as health care costs shift away from the fee-for-service paradigm, requiring new ways to deal with the small costs of care. Dematerialization further changes the paradigm by enabling care access outside of formal settings, such as through mobile health apps for therapeutics. Finally, democratization widens health care access thanks to these new characteristics.

Therefore, digital changes in the health care and public health sector need to be particularly mindful of 1 particular 6D [16] the creation of a digital divide due to a lack of democratization, meaning that digital changes can impose high hurdles to health promotion or health care access for subgroups of individuals with lower digital literacy or different language backgrounds. Very often, those are also individuals who may benefit most from prevention or health care. Such barriers can be overcome, for example, by making user interfaces as simple as possible and by providing personal support, which can also be assisted by digital means. Therefore, it is advisable to include equity aims in digital change processes, and to define the purpose of digital change as a “quadruple aim.”

### Examples of Leveraging Digital Advances in a Pragmatic Digital Public Health

Some digital advances in public health are already contributing toward making health care more equitable to underserved populations in low- and middle-income settings. Telemedicine, for example, enables patients to consult with health care providers remotely, breaking down geographic barriers to care. Digital technologies have also enabled health care professionals to reach more patients with chronic conditions, offering remote monitoring and management tools that improve patient outcomes while reducing costs. Bringing a spotlight on sub-Saharan countries, these innovations in health care access also were driven by the application of drones in delivering medicines, vaccines, and diagnostics tests to remote areas [41,42]. Furthermore, drones have been seen playing an important role in identifying malaria-vector breeding sites in rural areas as an important tool for disease surveillance [43].

As another example yet focused on low- and middle-income countries, Reach Digital Health [44] is leveraging digital technology to transform patient care, streamlining care requests, check-ins, follow-ups, and symptom assessments for a more patient-focused approach. Key initiatives include MomConnect [45], a WhatsApp smart bot that provides maternal and child health information in local languages to pregnant women in Sub-Saharan Africa, and Young Africa Live [46], a digital platform that empowers youth to make informed decisions about their sexual health. These innovative interventions, aimed at making health care more accessible and proactive, highlight the transformative potential of digital health in addressing public health challenges globally.

Expanding these digital advancements for a global health perspective, a notable instance of this progression is the “Global Flu View” platform [47]. This tool, which provides real-time tracking of global influenza activity based on participatory surveillance, signifies a transition from traditional to digital epidemiology. It presents unmatched opportunities for early detection, timely intervention, and strategic planning, all of which are crucial for managing influenza outbreaks and potential pandemics. Beyond its data visualization capabilities for influenza-like illness trends, Global Flu View also promotes knowledge sharing among public health practitioners [48]. As such, Global Flu View can also act as a template for similar collaborations in the field of NCD by acting as a code lab repository for exchanging scripts and algorithms for data analysis, facilitating peer collaboration, and fostering continuous learning and skill development of researchers and health care professionals. Furthermore, these digital platforms do not just address immediate public health needs; they also provide a valuable resource for training future digital epidemiologists. Through hands-on interaction with real-time health data, trainees can develop skills to transform intricate health data into actionable public health interventions.

### Emerging AI Trends and Their Possible Role in Digital Public Health

In addition to automating complex data analysis and predictions, AI-driven digital transformation can replace repetitive tasks in stable environments, such as administrative tasks and data entry [18]. This frees up public health professionals and health care providers to focus on personal interactions with citizens and patients. Along those lines, it is vital to identify which human interactions or tasks cannot be easily replaced by digital technologies and should be strengthened, such as counseling and emotional support [19]. AI can also provide citizens and patients with new tools to take a more active role in managing their health [49]. However digital health is not a replacement for doctors or other health professionals, but rather a companion to guide individuals through the health care system. By leveraging digital tools, citizens and patients can take a more active role in managing their health and accessing care. This can help to address some of the challenges associated with aging, such as chronic disease management, as well as promote health equity by improving access to care for underserved populations. Additionally, digital health can help to promote sustainability in health care by reducing the need for in-person visits and streamlining care delivery.

Recent advancements in large language models (LLMs) have the potential to greatly enhance public health interventions by providing tailored, accessible information at scale [50]. LLM technology facilitates the dissemination of health information in a way that can be easily adapted to a population’s education level, cultural background, or general preferences. This can be particularly beneficial in public health campaigns, where the goal is to effectively communicate with diverse audiences who may find traditional medical jargon intimidating or confusing [51]. LLM technology, through its natural language processing capabilities, allows for the analysis of population-level data, enabling the creation of public health recommendations and advice that are contextually relevant. It can help communities better understand their collective risk factors or prevalent health conditions, thereby promoting health literacy, preventive measures, and access to care. The capability of LLM to interact in a more human-like chat can also revolutionize how public health messages are communicated, making them more engaging and comprehensible. Moreover, with its ability to provide 24/7 service, LLM has become an efficient, ever-present tool for promoting lifestyle changes and mitigating noncommunicable diseases at the population level. There is already evidence from health care settings illustrating the benefits of LLM technology in enhancing patient interactions. Applying this technology on a broader scale could potentially have transformative impacts on public health communication strategies, risk mitigation, and health promotion. Given the long time horizon of emergence and living with chronic diseases, a knowledgeable, tireless, emphatic, efficient, and 24/7-available companion would be a highly welcomed tool to support lifestyle changes or health promotion to avoid NCDs.

The opacity of some AI models in how predictions are achieved (eg, in neural networks) is a common point of critique and reason for nonadoption. Explainable AIs enable public health experts, health care providers, and patients to understand how AI-based systems arrive at their decisions and recommendations [52-57]. This can help to address issues of bias and discrimination in health care and public health, which can arise when algorithms are based on flawed or incomplete data sets. By providing transparency and accountability, explainable AIs can help to ensure that health care automation is more equitable and unbiased, although this area is still underproof and requires more evidence to achieve its pledge [58,59]. Machine unlearning is another emerging area of research in digital health that has the potential to promote equity and fairness in health care automation [60-62]. Machine unlearning involves the intentional removal of certain data points or variables from a machine learning algorithm in order to correct biases or errors. For example, if an algorithm is found to be discriminating against certain patient populations based on race or ethnicity, machine unlearning can be used to remove those variables and correct the bias. By doing so, machine unlearning can help to ensure that health care automation is more equitable and inclusive.

However, while explainable AIs and machine unlearning can help to promote equity and fairness in health care automation, they also raise important questions about the role of humans, liability, and trackability in the digital health space. For example, who is responsible if an AI-based system makes a mistake or causes harm? How can we ensure that these systems are transparent, accountable, and auditable? And what role do humans play in overseeing and monitoring these systems? These problems are even exacerbated in any application of AI in public health decision-making or prediction at the population level. While problematic treatment decisions in health care will be spotted and corrected through close patient monitoring, similar monitoring for larger populations is much more complex. Furthermore, AI-driven public health decisions may impact specific population subgroups differently. Therefore, a decision of whether AI-supported decisions are good or bad requires a much more complex framework that includes considerations of equity, vulnerability, effectiveness, and efficiency. As such, it is important to consider not only the technical aspects of AI-supported public health, but also the social, legal, and ethical implications of these emerging technologies. By doing so, we can ensure that digital health remains an inclusive, equitable, and accessible space for all patients and that these new technologies truly serve as companions to guide individuals through the health care system.

Federated learning and swarm learning hold transformative potential for public health, providing a path to harness vast, diverse data sets while preserving data privacy and data ownership. Federated learning involves training machine learning models across multiple decentralized instances with a central trusted server [63]. This offers a powerful solution for public health systems grappling with the need to analyze large, varied data sets while respecting privacy concerns. Swarm learning, in contrast, uses a completely decentralized network of instances to collaboratively train machine learning models in a peer-to-peer manner [64]. This becomes especially valuable in public health contexts where data are highly decentralized and distributed across numerous devices and locations. Swarm learning allows public health practitioners to harness the power of distributed data to create more precise and effective machine learning models, ensuring data privacy and security. Adopting these approaches can have profound benefits for public health systems aiming to enhance their digital health initiatives [65]. By refining algorithms at the population level, as well as in specific subpopulations, public health systems can improve the precision of disease prediction and early detection. This also allows for the identification of opportunities for targeted interventions and treatments, which in turn can help reduce health care costs, enhance health outcomes, and promote health equity and accessibility. Moreover, federated learning and swarm learning provide tools for developing more effective public health policies and interventions. By analyzing patterns and trends in population-level health data, public health systems can identify high-risk groups and more effectively direct interventions. This ability can play a crucial role in preventing the spread of infectious diseases, reducing health care costs, and enhancing public health outcomes overall.

## A Pragmatic Vision for Making Inroads to Digital Change of Public Health for NCD

Digital change offers an unprecedented opportunity to address the substantial burden of NCDs at scale. However, the application of digital technologies needs to be thoughtfully aligned with public health functions, goals, and broader societal implications. Digital platforms can serve as tools for better public health monitoring, decision-making, as well as public engagement, and education. On the individual level, prevention requires behavioral changes and continuous personal efforts. One of the key downstream factors in a micro-level (individual) perspective is the psychosocial mechanisms mediated by social support, social influence, and social engagement [66]. Having these aspects addressed might mean a successful impact on the digital change of public health. On one hand, personalized prevention strategies have proven effective but lack scalability. On the other hand, digital solutions, such as telehealth platforms and health monitoring applications, may offer more scalable approaches by improving efficiency, effectiveness, and user experience.

While digital change opens new avenues, it simultaneously poses challenges related to equity and access. Populations that are (1) economically disadvantaged, (2) geographically isolated, or (3) digitally illiterate are at risk of being left behind in the digital transformation journey. Any initiative must include a comprehensive strategy to ensure that these changes do not exacerbate health inequities but rather work toward eliminating them. Furthermore, effective digital and nondigital NCD prevention at a population level demands a societal consensus. It often requires a willingness by people not directly at risk to participate in a larger social contract. For example, the legislation of smoking bans and “sugar taxes” may affect individuals who do not directly benefit from such measures. Therefore, stakeholders, from policy makers to health care providers and the general public, need to have realistic expectations about the benefits, potential harms, and costs of digital changes in health care or public health service delivery. Ongoing monitoring and evaluation mechanisms need to be in place to gauge the broader-scale implications of digital change.

### Conclusions

Digital health and AI in particular have great potential to make public health and health care of NCDs more efficient, effective, user-friendly, and equitable: the quadruple aim. Digital change in any of the 5 essential public health function domains should be purposeful and bring improvements in at least 1 dimension of the quadruple aim while not significantly compromising the other dimensions. But ultimately the success of digital public health and AI-supported public health measures will require clear criteria, monitoring, and benchmarks on how to achieve benefits for the majority while not putting minorities and vulnerable groups at a disadvantage. As such, AI explainability and unlearning of discriminatory or biased decision-making will be even more crucial for population-level decisions (which are common in public health, eg, regarding resource allocation) than in individual-level health care. Therefore, the close involvement of stakeholders, including patients, citizens, and vulnerable and minority populations, will be crucial in the development, roll out, and monitoring of AI-powered digital public health.

## Abbreviations

AI: artificial intelligence
DPT: digital proximity tracing
LLM: large language model
NCD: noncommunicable disease
